# Slicing on the Road: Enabling the Automotive Vertical through 5G Network Softwarization

**DOI:** 10.3390/s18124435

**Published:** 2018-12-14

**Authors:** Claudia Campolo, Ramon dos Reis Fontes, Antonella Molinaro, Christian Esteve Rothenberg, Antonio Iera

**Affiliations:** 1 Dipartimento DIIES, Università Mediterranea di Reggio Calabria, Via Graziella, Loc. Feo di Vito, 89122 Reggio Calabria, Italy; claudia.campolo@unirc.it (C.C.); antonio.iera@unirc.it (A.I.); 2 School of Electrical and Computer Engineering, University of Campinas (UNICAMP), Campinas 13083-872, Brazil; ramonrf@dca.fee.unicamp.br (R.d.R.F.); chesteve@dca.fee.unicamp.br (C.E.R.)

**Keywords:** network slicing, V2X, 5G, MEC, SDN, Mininet

## Abstract

The demanding requirements of Vehicle-to-Everything (V2X) applications, such as ultra-low latency, high-bandwidth, highly-reliable communication, intensive computation and near-real time data processing, raise outstanding challenges and opportunities for fifth generation (5G) systems. By allowing an operator to flexibly provide dedicated logical networks with (virtualized) functionalities over a common physical infrastructure, network slicing candidates itself as a prominent solution to support V2X over upcoming programmable and softwarized 5G systems in a business-agile manner. In this paper, a network slicing framework is proposed along with relevant building blocks and mechanisms to support V2X applications by flexibly orchestrating multi-access and edge-dominated 5G network infrastructures, especially with reference to roaming scenarios. Proof of concept experiments using the Mininet emulator showcase the viability and potential benefits of the proposed framework for cooperative driving use cases.

## 1. Introduction

The automotive vertical market is undergoing key technological transformations. Thanks to advancements in automation, sensing, and positioning techniques, self-driving vehicles are no longer science fiction but they are set to become a reality. Merging the local perception provided by on-board sensors, e.g., Global Positioning System (GPS), Light Detection and Ranging (LIDAR), Radio Detection and Ranging (RADAR), and cameras, of automated vehicles with information provided by other vehicles’ sensors and by the roadside infrastructure (e.g., about obstacles, road surface conditions, and planned maneuvers) will improve the perception of the surroundings and the overall vehicle safety. This is known as cooperative driving and includes both cooperative sensing and cooperative manoeuvring services, raising challenging demands in terms of coverage and connectivity in order to guarantee ultra-low latency (below 10 ms), ultra-high reliability (near 100%) and high data rate (in the order of Gbps) under high mobility and density conditions.

Such strict demands cannot be met by current technologies, neither the IEEE 802.11 variants nor the Long Term Evolution (LTE); this is why the research and industrial communities have struggled to come out with the specifications of a new technology, which has a clear and forward compatible evolution path towards the fifth generation (5G) system.

Efforts toward this direction are being promoted by key players in the automotive and telecommunications industries within the 5G Automotive Association (5GAA) initiative [1]. The Third Generation Partnership Project (3GPP) specified a set of technical enablers in Releases 14 and 15 to provide a spectral-efficient air interface, with a focus on localized vehicle-to-vehicle (V2V) communications. The new technology, called Cellular V2X (C-V2X), targets advanced driving assistance services, such as cooperative driving [2] and platooning [3].

Such results alone cannot fully address the challenges of the V2X ecosystem. The entire end-to-end chain of radio, networks, applications and (cloud) services of upcoming 5G systems requires customization and architecture enhancements to meet the highly demanding V2X performance requirements, currently under debate within 3GPP Release 16 [4]. For this reason, *network slicing* has been recognized by the standardization bodies and the research community as a prominent solution to fulfill the stringent V2X requirements [4,5,6].

By logically isolating control plane (CP) and user plane (UP) network functions (NFs), network slicing [7] can tailor resources to specific vertical markets’ needs on a common programmable network infrastructure. This is viable through network softwarization technologies [8] such as Network Function Virtualization (NFV) [9] and Software-defined Networking (SDN) [10], and through Multi-access Edge Computing (MEC) principles [11]. Thanks to NFV, UP and CP functionalities of different slices can be virtualized, independently scaled and displaced in convenient locations to flexibly support various services. For instance, UP functions can be located close to the user, in edge cloud facilities, by exploiting MEC deployments [12] to reduce service access latency. Edge nodes can also be exploited by verticals and over-the-top providers for storage, processing and dynamic service creation within a given network slice, thus introducing another multi-tenancy dimension [13]. SDN allows the remote configuration of the physical network to reserve on demand networking resources for the slices and to steer network traffic accordingly [14].

A set of references slices are typically considered, where each of them represents categories of use cases identified by the International Telecommunication Union (ITU) [15] and by the Fifth Generation Public–Private Partnership (5G-PPP) [16], i.e, enhanced mobile broadband (eMBB), massive machine-type communications (mMTC), and critical communications (CriC), also known as ultra-reliable and ultra-low latency communications (URLLC). A slice for eMBB services requires very high data rates to ensure access to multimedia contents, services and data such as ultra-high definition video streaming and augmented reality. A slice supporting mMTC should sustain the traffic load generated by massively connected devices (e.g., sensors enabling smart home and smart building monitoring), typically transmitting a relatively low volume of non-delay-sensitive information. mMTC traffic is mainly concentrated in the Radio Access Network (RAN) segment, thus most of the information flow should not pass through the core network both for security and communication efficiency. Reliability, low-latency and security will be critical for the URLLC slice to provide services that are extremely sensitive to latency such as emergency services and tactile Internet. They would greatly benefit from dedicated functions instantiated at the edge.

The need for a dedicated V2X slice has been early argued in [6], and is currently under debate in 3GPP [4]. Indeed, the unique challenges of the vehicular environment [17] and the peculiarities of V2X services do not easily allow a straightforward mapping into the aforementioned reference slices. The major V2X issues that need to be addressed when designing a network slicing framework supporting the automotive vertical are the following:

- Localized broadcast communications and edge-dominated access should be considered as crucial slice behaviors to support cooperative driving applications demanding ultra-low latency and ultra-reliable connectivity over the RAN.
- Besides safety applications, having as ultimate goal autonomous driving, multiple heterogeneous services with diversified requirements should also be simultaneously supported (e.g., high-bandwidth infotainment services and diagnostics services) pushing for strict slice isolation.
- The high mobility of vehicles makes frequent roaming events calling for multi-operator slice coordination. To guarantee a consistent user experience in case of roaming, a basic set of functionalities of a V2X slice that cover the main requirements (e.g., mobility and authorization/authentication) should be agreed among operators and proper interfaces conceived for their interactions. In so doing, slices composed of the same network functions/behaviors could be available for the user when moving to a visited network, while ensuring service continuity.

In such a challenging context, this paper aims at providing the following main contributions:

- Design of an architectural framework to support V2X network slicing, aligned with the latest 5G specifications. The framework neatly points out the role and impact of the MEC paradigm and the potential of its interplay with other network softwarization technologies, i.e., SDN and NFV. The behavior is analyzed with special focus on the roaming procedures for a V2X slice supporting the challenging cooperative driving use case.
- Experimentally demonstrate the capabilities of a programmable, multi-access, edge-dominated network infrastructure to support V2X slices, through Proof-of-Concept (PoC) experiments based on the Mininet-WiFi [18] wireless SDN emulator. We overhauled Mininet-WiFi to resemble the main components and mechanisms of the conceived network slicing framework. The results of the reproducible experiments point to the viability of the proposal and the potential benefits.

The remainder of the manuscript is organized as follows. In Section 2, background material for our work is provided, while scanning the related 3GPP C-V2X and 5G specifications and the literature on network slicing for V2X. Section 3 provides a general overview of the proposed network slicing framework. Details about the design of a V2X slice for cooperative driving on top of the conceived framework are given in Section 4. Section 5 reports the description of the mobility management procedures in the proposed framework. A reference use case is practically showcased in Section 6, where achieved results are discussed. Finally, Section 7 summarizes concluding remarks and arguments about future works.

## 2. Background, Motivation, and Contributions

### 2.1. From Cellular-V2X to the 5G Service-Based Architecture

3GPP is pushing the C-V2X technology as a solution enabling vehicles to communicate with everything, i.e., other vehicles (V2V), vulnerable road users (vehicle-to-pedestrian, V2P), roadside infrastructure (vehicle-to-infrastructure, V2I), and the cloud/edge servers (vehicle-to-network, V2N). The technology uses the cellular network infrastructure to provide service to vehicular user equipments (VUEs) and enable enhanced direct V2V connectivity (be it network-assisted or unassisted) through the PC5 sidelink interface [19,20]. The following entities have been added to extend the cellular architecture in Release 14 [21] and to specifically manage V2X communications:

- The V2X Control Function is the logical function for V2X network-related actions. It provides configuration parameters for VUEs located under the coverage of an eNodeB as well as out-of-coverage, i.e., without the cellular infrastructure service.
- The V2X Application Server (AS) has a wide range of functionalities including: the reception of uplink unicast data from the VUEs; the delivery of data to the VUEs in a target area using unicast and/or multicast interfaces. It may also provide centralized control and distribution of traffic, road, and service information.

The C-V2X standard is still evolving and it is expected to embrace upcoming 5G specifications to support more advanced driving assistance services. The latter ones are classified by 3GPP in four categories [22]. Vehicles platooning, which dynamically forms a group of vehicles travelling together at short inter-vehicle distances. Advanced driving, which we call here cooperative driving, enables vehicles to share local sensor data and driving intentions with vehicles in proximity, thus coordinating trajectories and maneuvers. Extended sensors services, which foresee the exchange of raw/processed sensor data or live video among VUEs, RSUs, pedestrians and V2X ASs. Remote driving, which allows a remote driver or a cloud application to tele-operate a (private or public) vehicle; this is useful for those passengers who cannot drive themselves or when the vehicle is located in dangerous or uncomfortable environments.

**Figure 1:**
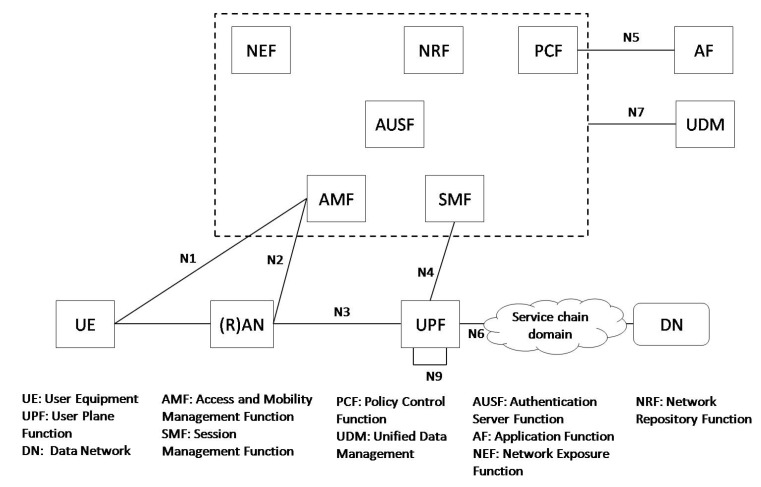
The 5G service-based architecture.

The 5G architecture, recently specified by 3GPP [23], is natively conceived with softwarization in mind. The Core Network (CN) consists of various NFs, with a clear separation of CP functions and UP functions (UPF), as depicted in Figure 1. The Access and Mobility Management Function (AMF) provides UE-based authentication, authorization, mobility management, etc. The Authentication Server Function (AUSF) stores data for authentication of UEs, while the Unified Data Management (UDM) stores UEs subscription data. The Session Management Function (SMF) is responsible for session management and also selects and controls the UPF for data transfer. The Policy Control Function (PCF) may instruct different routing policies.

To specifically address the cloud-native design and the paradigm shift from a network of entities to a network of functions, the following functionalities have been designed. The Network Repository Function (NRF) provides registration and discovery functionality, allowing NFs to discover each other and communicate via open Application Programming Interfaces (APIs), in contrast with LTE that uses predefined interfaces between the elements. For instance, the AMF service exposes to other NFs the information regarding mobility-related events and relevant statistics [24]. The Network Exposure Function (NEF) provides the means to collect, store and securely expose the services and capabilities provided by 3GPP network functions (e.g., to third parties or amongst NFs themselves). The Application Function (AF) represents any additional CP functions which might be required, e.g., to implement network slicing.

3GPP is investigating in [4], as part of Release 16, potential architecture enhancements of the 5G system design to support advanced V2X services. Among them, there is the network slicing concept, flexibly enabled on top of the new 5G architecture.

### 2.2. Network Slicing for V2X Communications

Addressing the demands of V2X applications entails a holistic approach that goes beyond the valuable ongoing effort of improving connectivity over the RAN. This is why the network slicing concept comes into the picture, with a solution that can potentially exhibit an end-to-end (E2E) scope encompassing both the RAN and the CN segments of a 5G system. Indeed, a slice can span all domains of the network: software modules running on cloud nodes, specific configurations of the transport network supporting flexible placement/migration of functions, a dedicated radio configuration or even a specific Radio Access Technology (RAT), as well as configuration of the device.

The potential of the network slicing concept for the vehicular environment and the need for *dedicated* V2X slicing has been early unveiled in [6] and then further elaborated in [25], where a set of guidelines for the design of a network slicing V2X framework are dissected. Embracing the vision of V2X tailored slicing, in [4], 3GPP mentions the usage of customized parameters identifying a slice for the automotive industry. More in detail, a specific *customized* and *standardized* Slice Service Type (SST) is proposed within the Network Slice Selection Assistance Information (NSSAI) parameters [23]. The SST refers to an expected network behavior in terms of features and services as requested by a given slice. The NSSAI also includes a Slice Differentiator (SD), that can help selecting among several network slice instances of the same type, e.g., to isolate the traffic related to different services provided over different slice instances. A standardized value could facilitate roaming support; it can be expected that vehicles move quickly and often cross over different countries/Public Land Mobile Networks (PLMNs) while connected to the network.

The V2X umbrella term actually covers a multiplicity of use cases characterized by diverging service and connectivity requirements. Indeed, with the penetration of self-driving vehicles which require no human involvement, humans inside the vehicle will engage themselves in other more relaxing activities such as media consumption (e.g., web browsing or video streaming), reading, and sightseeing. This feature would further suggest the design of customized network slices for different V2X services. The autonomous driving, tele-operated driving, vehicular infotainment, and vehicle remote diagnostics and management slices are proposed in [6].

Elaborating on the idea of different slices for different V2X services, the work in [26] focuses on the resource allocation, in the RAN only, for the autonomous driving slice and the infotainment slice as two sub-slices created on a common physical infrastructure to provide isolated services. The Road-side Unit (RSU) provides infotainment services only and decides which vehicle will act as a relay for the autonomous driving slice based on V2I and V2V link quality. These relays, acting as virtual Access Points (APs), are responsible for the reliable exchange of safety messages between vehicles of the autonomous driving slice. Further works analyze, at a high level, how to apply the network slicing concept to the V2X environment [27,28].

To validate the theoretical design of network slicing solutions for V2X, experimental evaluations are important to shed light on their performance. Few field tests have been carried out thus far; for example, in November 2016, SKT and Ericsson jointly deployed network slicing on a 5G radio network infrastructure (operating on mmWave frequency bands) in a BMW car test track in South Korea [29]. Clearly, performing real car tests incurs high cost and entails legislation issues, while making hard the reproducibility of results. A simpler and cheaper playground can be very helpful to preliminarily investigate technical solutions before field tests, as targeted in this work.

Lately, some tools have been proposed to analyze network slicing without having to wait for the ratification of the 3GPP 5G standards. Among them, the open-source OpenAirInterface (OAI) platform [30] is a software implementation of the LTE protocol stack and can operate over commodity hardware for the deployment of the eNodeB and the CN. OAI has been exploited in conjunction with other platforms (e.g., FlexRAN and LL-MEC) in [31] to showcase the benefits of the coordinated programmability spanning RAN and CN domains to facilitate different V2X slices, i.e., remote diagnostics and management and vehicular infotainment slices, as defined in [6]. However, there, no details are provided about the main entities and functionalities required to manage the considered V2X slices.

### 2.3. Contributions

The surveyed literature clearly shows that, overall, the topic of network slicing for the automotive vertical is still in its infancy. On the one hand, its potential has not been adequately disclosed and a clear specification of the required V2X customization in the 5G slicing architecture are still missing; on the other hand, practical design has been marginally targeted in the literature. To fill these gaps, this paper aims to advance the state of the art with the following main contributions:

- A 5G-V2X network slicing framework is designed that enables the interplay of the MEC, SDN and NFV paradigms for the automotive vertical context. The high-level structure is inspired by a widely accepted three-layer architecture, but we make the effort to specify and customize to the V2X case the involved entities, their roles, and the mechanisms required to orchestrate the lifecycle and the behavior of V2X slices with a focus on the cooperative driving service.
- Special attention is given to the way of handling cross-operator interaction that is crucial for V2X due to mobility of vehicles. Specifically, the concept of federated slicing is considered vital to manage the roaming procedures for the V2X slice.
- Some reproducible PoC experiments are implemented through the Mininet-WiFi [18] wireless SDN emulator, which has been overhauled to demonstrate the designed framework capability of supporting the challenging case of cooperative driving in a multi-operator environment.

## 3. 5G-V2X Network Slicing Architecture

In this section, we present the proposed 5G-V2X network slicing high-level architecture. Although there is no single design available of a unified framework that supports the network slicing concept [5,13,28,32], there is a wide consensus on a *three-layer* model [28] plus an additional management and orchestration layer, which neatly identifies the involved players and domains. Without loss of generality, our proposal builds upon the model described in [28], which includes the following layers: the Infrastructure layer, the Service layer, and the Business layer. The three layers encompass components that are aligned with the 5G service-based architecture and its key enablers, i.e., SDN, MEC, and NFV. Within the conceived general design of the three-layer architecture, we customize specific functionalities and procedures to support V2X slicing, based on the guidelines we identified in [25], and summarize in Table 1.

### 3.1. Architecture Components

**Figure 2:**
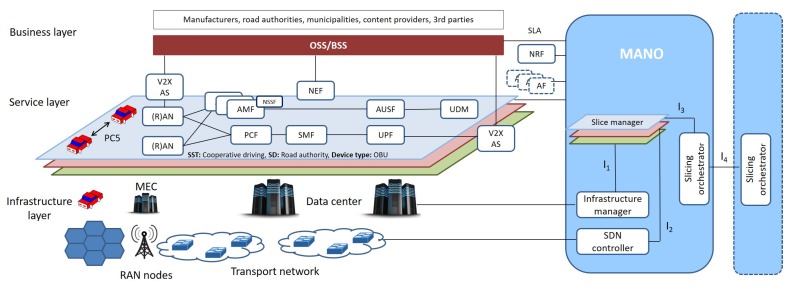
Network slicing framework and slice instantiation for cooperative driving services.

The three layers of the high-level architecture and the relevant components are illustrated in Figure 2 and briefly discussed in the following.

The Infrastructure layer includes RAN nodes and devices, MEC servers, and the Transport network.

**Figure 3:**
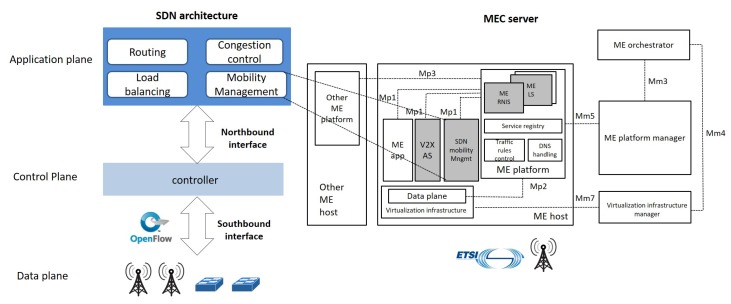
SDN architecture with main network applications (**left**) and MEC server deployment (**right**).

- RAN Nodes and devices. Heterogeneous RAN technologies characterize the V2X scenario, including Wi-Fi APs, RSUs, and eNodeBs. We consider all network devices as SDN-enabled switches managed by SDN controllers.
- MEC servers. They host network functionalities and applications that require ultra-low latency access. We refer to the European Telecommunications Standards Institute (ETSI) framework, as a reference for their deployment [33]. There, the MEC server is referred to as Multi-access Edge (ME) host [33]. It is an entity that contains the ME platform and a virtualization infrastructure which provides compute, storage, and network resources for the ME applications. The latter are running, e.g., as virtual machines (VMs) or containers, on top of the virtualization infrastructure provided by the ME host. They can interact with the ME platform to consume and offer ME services. To match the requirements of delay-critical and/or bandwidth-demanding V2X slices, we propose to deploy the V2X AS, normally placed in remote clouds, as an ME application running in the ME host. The ME services Radio Network Information Service (RNIS) and Location Service (LS), envisioned by ETSI [33] (see Figure 3, right), can also play a role for V2X slicing by providing radio-related information, i.e., low-level signaling, helpful to provide radio-related augmented services and to determine the location of a connected device.
- Transport Network. The SDN-enabled transport network interconnects CP and UP Virtual NFs (VNFs) and Physical NFs (PNFs), in the RAN and CN, among each other and with external data networks. SDN is used to steer traffic between NFs of a given slice through the set-up of paths that can be automatically reconfigured either to handle traffic engineering requirements or to react to possible network failures and changing conditions (e.g., mobility).

The Service layer hosts the 5G network functions [23], which can be PNFs or VNFs deployed by the operator and/or by a third party to enforce a given network behavior in the slice. Following NFV principles, UP and CP functionalities of different slices and be independently scaled and displaced in convenient locations and then chained through SDN to flexibly support various and novel services.

The Business layer resides at the top of the model and includes services and use cases of the vertical markets for which slices are designed. It encompasses the mechanisms and tools to describe the slice behavior at a high level, and to capture the requirements of a given Service Level Agreement (SLA) for a vertical segment, whose assurance is tracked by the Operation Support System (OSS)/Business Support System (BSS). Such a description needs to be then translated to the underlying layers.

The Management and Orchestration (MANO) realm includes a Slice Manager, a Slice Orchestrator, an SDN Controller, and an Infrastructure Manager.

- Slice Manager. It operates at a *per-slice* level and is responsible for: *(i)* slice description through well-defined templates; *(ii)* slice instantiation, which encompasses the identification of CP/UP architecture, interfaces, slice-specific and common VNFs/PNFs, and RAN/CN/device parameter settings; and *(iii)* slice life-cycle management, which entails slice configuration, adaptation and monitoring to fulfill isolation constraints and agreed SLAs.
- Slicing Orchestrator. It enables the brokering of resources (hosted in the device, at the edge, on the network and on the cloud) among multiple slices of the same operator. It interacts with the NRF to build the service/network chains by leveraging off-the-shelf NFs and additional AFs, if required. V2X services, especially safety ones, should be supported over unfettered geographical coverage with available resources irrelevant of the specific operator. Thus, it is vital that the slicing orchestrator also exchanges information with peer entities of other Mobile Network Operators (MNOs) to enable seamless platform sharing among operators at a global scale for continuous and guaranteed user experience, according to the *federated network slicing* concept, under discussion within different standardization bodies (e.g., 3GPP [34] and GSMA [35]) and particularly challenging for V2X due to frequent roaming events.
- SDN Controller. Network resources are managed by logically centralized and physically distributed SDN controllers (Figure 3, left) dealing with traffic engineering functions, network statistics monitoring and implementing southbound interface protocols, e.g., OpenFlow (OF) [36], to install flow-level rules in SDN-capable nodes. Vehicle mobility poses serious issues to the run-time configuration of a slice. Quick path resource allocation algorithms in the backhaul/fronthaul segments are needed to allocate network resources and migrate NFs in another area, while reducing disruption periods. With MEC servers, live migration and service redirection are necessary actions, which further increase the mobility management and handover complexity [37]. To this purpose, a SDN *mobility management* application becomes crucial to collect radio-related data (e.g., received signal strength), the load status of each RAN node, and information about the position/trajectory of each vehicle, as provided by ME services. To ensure low-latency operation and facilitate the interaction with the ME services, the SDN mobility management network application can be transferred to the MEC server, as also suggested in [37]. Such a design choice, illustrated in Figure 3 (right), emphasizes the potential of the synergy between MEC and SDN in coping with the V2X challenges. Through the collected information and the up-to-date network state, the mobility management application can trigger: *(i)* seamless UP/CP functions migration (also their pre-fetching, whenever possible); *(ii)* network resources reconfiguration in the active slice(s); and *(iii)* handover procedures, e.g., according to the actual load status of each cell, instead of only considering Received Signal Strength Indicator (RSSI).
- Infrastructure Manager. It is in charge of orchestrating the infrastructure layer, by monitoring the processing and storage status of edge and cloud servers. To this purpose, it can build upon the ETSI NFV framework [38].

### 3.2. Interfaces

Four interfaces are defined within the MANO layer to facilitate slice orchestration and management, as shown in Figure 2.

- Interface $I_{1}$. First, information about the available computing resources are exposed through such interface by the Infrastructure Manager to each Slice Manager. Second, it serves the configuration of such resources as demanded by each Slice Manager.
- Interface $I_{2}$. It serves the monitoring of available network resources per slice, as tracked by the SDN Controller, and the specification of network requirements by each slice.
- Interface $I_{3}$. It exposes the per-slice requirements to the Slicing Orchestrator and the decision about reserved resources to each slice.
- Interface $I_{4}$. It allows the Slicing Orchestrator to interact with its peers of other MNOs.

Concerning the interfaces between other modules in the architecture, the proposal relies on the off-the-shelf 5G service-based architecture and the open APIs already provided there.

## 4. V2X Slice Design

On top of the described framework, different slices can be instantiated and orchestrated. Despite the heterogeneity of V2X services and applications (remote driving, vehicle platooning, infotainment, vehicle diagnostics, etc.), we identify a common set of minimum functionalities that should be provided, and possibly agreed by all operators, to a V2X slice to simplify the slice roaming management:

- AMF instances should be available regardless of the specific offered V2X service, to support vehicle mobility. Multiple dedicated AMF instances need to be flexibly deployed to avoid the AMF to be overloaded with consequent increase in latency during slice selection/attachment procedures (being such procedures managed at the AMF), while ensuring isolation with other (non-V2X) slices leveraging the same functionalities but less aggressively (e.g., pedestrian/indoor UEs).
- AUSF, PCF and UDM should be deployed as NFs, which are common to all V2X slices.

Besides such basic functionalities characterizing a *default* V2X slice, different functions can be dynamically chained and different behaviors can be flexibly configured to meet the specific performance requirements of a given V2X service category. In this work, we focus on the instantiation of a V2X slice for cooperative driving services. Such a choice is due to the relevance of such services in the roadmap towards connected and automated vehicles, and its challenging connectivity and computing demands. In particular, the cooperative driving slice should guarantee:

- Ultra-reliable and ultra-low latency V2V communications. V2V communications are leveraged to support the reciprocal exchange of vehicles’ kinematics parameters, the perceived environment and intended maneuvers. Dedicated bandwidth can be allocated to V2V links (e.g., 10 MHz as assumed in [19]) to ensure the isolation from other non-V2X slices in the RAN. A common unlicensed spectrum, i.e., the 5.9 GHz Intelligent Transportation Systems (ITS) spectrum, should be used for V2V communications to ensure cross-operator availability.
- V2N communications with the V2X AS. The following data need to be exchanged via V2N links with high reliability and low-latency: (i) raw sensory data sent by vehicles to the infrastructure for the purpose of 3D-map processing of the surrounding area and buildings; and (ii) the computed result sent in the opposite direction to extend the spatial vehicle perception [39]. Therefore, processing shall be carried at an edge cloud level. Multiple RATs and high-throughput connectivity options (e.g., mmWave links) could be used to boost the RAN throughput.

The resulting chaining of NFs for the cooperative driving slice is illustrated in Figure 2.

Slice identification and attachment. The proposed framework is compliant with the guidelines under discussion within 3GPP for what concerns the main operations related to the slice lifecycle. Granted the general slice configuration, for the sake of completeness, the slice attachment procedure [40] is shortly recalled here and customized to the specific case of a connected vehicle. A VUE, wishing to attach to a slice, provides the NSSAI to the network to enable the selection of a slice instance for it [23]. For the V2X slice of interest, the NSSAI parameters can be set as follows: the SST is set to *Cooperative driving*, the SD is set to the *Road Authority name*. On receiving a request from the On-Board Unit (OBU), the Network Slice Selection Function (NSSF), co-located with the AMF, performs the slice selection procedure by leveraging additional information, e.g., about the *Device type*, which in the most general case can be set as OBU, smartphone of pedestrians. Once the NSSF has checked the subscriber information and the VUE is authenticated, through interactions with the AUSF and the UDM, the slice attachment procedures are performed and the services can be accessed.

## 5. Mobility Management

In the context of V2X, mobility should be handled as a norm; it becomes a complicated issue in the case of roaming, requiring inter-operator interactions. We now focus on the capability of the proposed framework to manage inter-operator mobility, since intra-operator mobility can be seen as a simplified roaming case. Whenever a vehicle moves from the home network to a visited network, the same service quality must be seamlessly provided to it, if the federated network slicing concept is in place. This is possible thanks to the orchestration capabilities of the conceived network slicing framework, which allows tracking the vehicles and monitoring: *(i)* the link and quality availability in the end-to-end chain of radio and core network elements; and *(ii)* the processing capabilities of MEC servers, so to promptly trigger slices reconfiguration.

**Figure 4:**
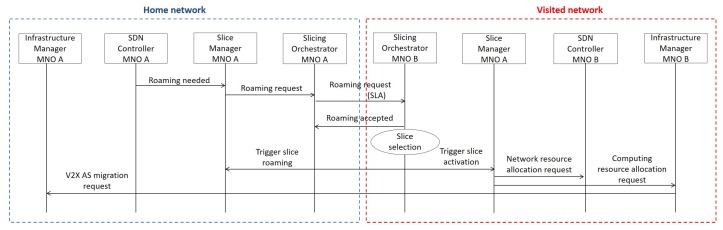
Main required interactions for the roaming procedure.

The main proposed interactions during roaming are depicted in Figure 4. The SDN Controller continuously monitors the status (i.e., position, planned trajectory, and radio coverage options) of currently served vehicles. Status parameters are retrieved from the MEC server and used by the SDN Controller to predict the need for a vehicle to roam from a home to a visited network. Whenever the SDN Controller detects that such a condition is going to occur, it notifies the home network Slice Manager, which interacts with the Slicing orchestrator. The latter one contacts its peer in the visited network to exchange the SLAs of the roaming slice.

The Slicing Orchestrator in the visited network checks, among the activated slices, the one that better matches the requirements of the slice to be hosted. If there is a slice that matches the indicated requirements, it is allocated to host the new customer. Otherwise, the slice that better suits the demands is adapted to accommodate the requirements of the visiting subscriber. In its turn, the Slicing orchestrator in the visited network may also resort to network resources from other operators (if slicing federation is in place) to ensure the desired service quality. In the worst case, a *default V2X slice* is activated for it, which ensures the basic functionalities.

The Slice Manager of the selected visited slice interacts, via the Slicing Orchestrator, with the Slice Manager of the home slice to agree about the overall slice configuration, e.g., whether and which UP/CP functionalities should be migrated, and triggers the release/activation of functionalities in the home/visited slice, respectively.

If the accessed service is available at a MEC server, the Infrastructure Manager of the visited network triggers a migration request for the V2X AS into the targeted MEC server. The MEC application migration between edges of different MNOs, with service continuity guaranteed, is still an open issue [41]. According to the available processing capabilities at the edge and the service requirements, the Infrastructure Manager could either decide to trigger a scale-up operation in the edge server and/or enforce cooperation among nearby edge servers [42], if the closest MEC server is overloaded.

The SDN Controller sets-up the paths to ensure a quick service migration and configures the RAN to guarantee a comparable quality (e.g., by instructing multi-RAT access). In the case of intra-operator mobility, the same procedures are triggered, with the main difference being that the Slicing Orchestrator is only required to interact with the Slice Managers of the slices activated by the same MNO to assure slice isolation in case of slice reconfiguration on the fly.

## 6. Proof-of-Concept Experimental Evaluation

We now turn our attention to the practical realization of a reference V2X network slice based on the Mininet-WiFi network emulator [18]. Mininet-WiFi is a fork of the popular Mininet SDN emulator and allows fast prototyping and experimental evaluation of wireless networks, including OpenFlow-enabled wired switches and wireless APs. The emulation of the wireless medium is supported by well-known propagation models. More details on the implementation and capabilities can be found in the documentation (e.g., user manual) available in the project repository [43].

Despite experimenting with 802.11 and not an actual 5G protocol suite, the wireless emulation and SDN control features of Mininet-WiFi allow valuable PoC experiments on a subset of the functionalities of the envisioned framework on top of programmable, multi-access, and edge-dominated network infrastructures, as previously presented in [14,42,44]. In particular, the functionalities required for V2N connectivity of a vehicle with a V2X AS were emulated, due to the end-to-end scope and relevance in the case of cross-operator mobility. Further implementation details are discussed next.

The main objective of the PoC experiments was to preliminarily showcase the viability and potential benefits of particular aspects of our V2X slicing framework proposal, considering off-the-shelf tools and technologies. More specifically, we explored the use cases of slicing federation in two different multi-operator reference scenarios to demonstrate, respectively: (*i*) slice capacity aggregation; and (*ii*) load-aware slice handover decision. The results from the experimental evaluation shed light on the potential V2X slice isolation and performance gains when operators agree to share resources through a proper slicing framework.

### 6.1. Implementation Choices and Details

All source code to reproduce the experiments and a figure of the software prototype architecture are available in our project repository [43]. The following implementation choices were considered to emulate the V2X environment:

- Vehicles were emulated as multi-interface wireless hosts capable of connecting to both eNodeBs and APs/RSUs.
- Heterogeneous RATs were modeled by configuring range and data rates of (R)AN nodes so to resemble either eNodeBs or Wi-Fi APs/RSUs, deployed by different MNOs as OF-capable switches, connected to the backbone and to nearby MEC servers.
- V2X ASs can be hosted either at a MEC server or in remote cloud facilities. In both cases, they were modeled as wired hosts.
- A Remote Authentication Dial-In User Service (RADIUS) server protocol (FreeRadius provides a multi-protocol policy server, supporting RADIUS, DHCPv4 and VMPS, and is available online: https://freeradius.org/) was used for Authentication, Authorization, and Accounting (AAA) purposes, resembling the behavior of the NSSF when interacting with the AUSF and UDM to check subscription information during the slice attachment procedures.
- As for the SDN Controller, Ryu (It was chosen due to Python programming friendliness and OpenFlow 1.3 support, including metering functionality to measure and control the rate of packets allowing to enforce rate limiting for roaming users; the code is available at: https://github.com/ramonfontes/ryu) was properly extended to run the network slicing and mobility management applications conceived to support V2X. More specifically, it processes radio control information by vehicular devices and tracks their connectivity and position. Based on collected information, the V2X slicing SDN application triggers the service migration (Please, note the simplification compared to the theoretical framework, where this functionality is implemented by the Infrastructure Manager) and configures the interfaces/links to be used for endpoints communication.

In the first reference scenario, we evaluated the capacity aggregation with a single SDN controller implementation tracking resources spanning the domains of different MNOs, i.e., converging the functions of the Slice Managers and Slicing Orchestrators along the roaming procedures of the proposed framework. By receiving RADIUS messages, the SDN controller tracks the user subscription information and enforces slice policy rules accordingly.

In the second reference scenario, in turn, we evaluated the load-aware handover decision with and without slicing federation, where different SDN controllers handling network resources (in particular RAN nodes) belonging to different operators share information such as RSSI and load status. With this information, controllers trigger handover actions to improve the slice performance stability and provide low latency.

### 6.2. Reference Scenario 1: Slice Capacity Aggregation

**Figure 5:**
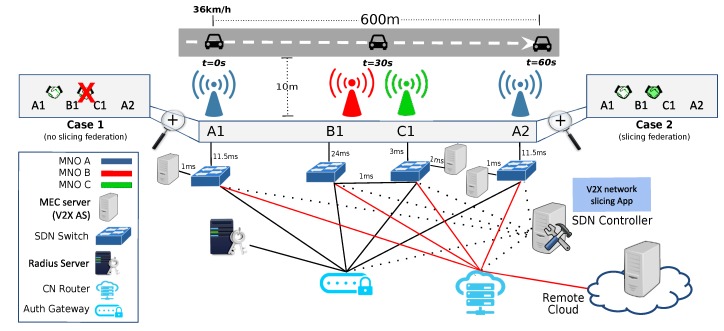
PoC reference scenario of slice capacity aggregation implemented in Mininet-WiFi.

Figure 5 illustrates a vehicle moving at constant speed (36 km/h) on a straight road (600 m long) connected to a V2X slice for cooperative driving services. The vehicle relies on V2V and V2N connectivity provided by its Telco operator (MNO A). The V2N link is used to connect to the V2X AS, wherever placed as previously discussed.

Initially (t=0), the vehicle is connected to an eNodeB (A1) of its home operator (MNO A) and the V2X AS is hosted by the corresponding MEC server. During the journey, the vehicle loses the coverage of its operator and it roams (e.g., due to a territorial crossing or a blind spot coverage) to another operator (MNO B) by attaching to eNodeB B1.

We assumed that other slices are currently supported by MNO B (e.g., for mMTC services which heavily load the access network), and the penetration of cooperative connected vehicles in the MNO B’s subscribers basis is low, thus a V2X slice satisfying the user requirements is not available.

Two cases can be explored at this point:

- Case 1 (No slicing federation). MNO B accepts the roaming vehicle and allocates a *default* V2X slice with the available resources, without disrupting the customer active slices but at the expenses of poorer quality. Since MNO B does not host the V2X AS in its MEC facilities, the vehicle is forced to connect to a remote V2X AS, and, hence, experiences larger latencies. In particular, the SDN controller handling the subscription information of the roaming vehicle (as tracked by the RADIUS server) applies the corresponding datapath (OF metering) rules to limit the bandwidth to 5 Mbps on the path from the vehicle to remote V2X AS (i.e., across B1 and the core network).
- Case 2 (Slicing federation). MNO B does its best to guarantee to the roaming vehicle the same quality as in the home network by means of a federated network slicing approach including MNO C resources. Multi-connectivity access over the (R)AN is activated to boost the throughput performance by bonding the radio interfaces as triggered by the SDN Controller configuring the corresponding flow tables, when the overlapping coverage areas of B1 and C1 are detected.

In both cases, after some time, the vehicle returns to its home MNO through A2. Note that, for the sake of implementation simplicity, a single cell in the network of MNO B is traversed by the vehicle before switching back to the coverage of MNO A.

**Figure 6:**
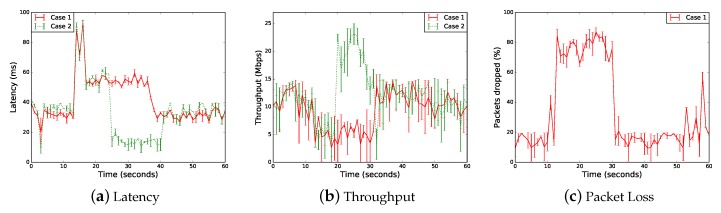
Experiment metrics measured when the vehicle travels outside the reach of eNodeB A1 towards A2 through the coverage B1 and A2 for Case 1 of no slicing federation and Case 2 with federation.

The benefits of slicing federation in terms of the impact on measured latency and throughput of the vehicle when accessing the V2X AS through different MNOs can be seen in Figure 6a,b, which presents the results of 10 experiment runs. When slicing federation is in place (Case 2), the vehicle experiences better performance. Federation enables multi-RATs access so that the vehicle also connects to C1 and benefits from higher throughput. Sudden increasing throughput demands by the vehicle may raise to exchange data at an intersection, where the visibility may be obstructed and the MEC server may be particularly helpful to build the 3D-surrounding maps. The measured latency is significantly lower (compared to the non-federation Case 1) and the service disruption is reduced when the vehicle is served through the federated MNO C, also thanks to the pro-active migration of the V2X AS at the edge of MNO C. The viability of the pro-active migration of applications among edge nodes is investigated in [45,46].

Figure 6c shows the effectiveness of OpenFlow 1.3 metering rules in B1 to drop packets identified as MNO A customer exceeding 5 Mbps in Case 1.

### 6.3. Reference Scenario 2: Load-Aware Slice Handover Decision

**Figure 7:**
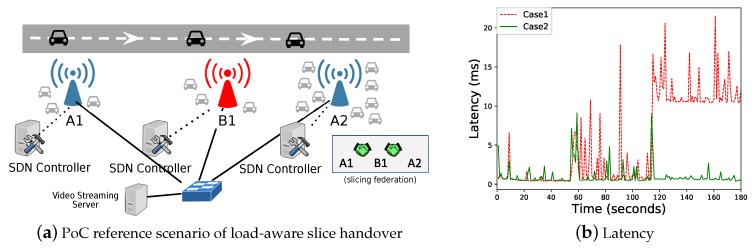
Latency perceived by the targeted vehicle with and without slicing federation (**b**) in the reference scenario shown in (**a**).

Figure 7a presents the reference topology for the second investigated scenario intended to show the effectiveness of slicing federation under a multiple SDN controller setup. The target vehicle moves from eNodeB A1 towards eNodeB A2 and stops between eNodeB B1 and A2. When federation is not in place (Case 1) between the two operators, MNO A and MNO B, the controller tracking the vehicle has no knowledge about the availability of slice connectivity options providing higher quality, i.e., the less loaded eNodeB B1. Thus, following a traditional RSSI-based handover, the vehicle ends up associating with A2, contributing to a further loaded eNodeB at risk of service degradation. Differently, when the federated network slicing concept is in place (Case 2), knowing that A2 is overloaded, the SDN controller does not trigger the handover to A2. Through this load-aware slice handover strategy, slice-specified quality levels, such as those affected by base station load, can be provided. In this use case, the controller for MNO A, through the federated information exchange with its peer from MNO B, discovers the availability of resources provided by MNO B in the area where the vehicle moves. Despite being in range (and eventually better RSSI) of A2, after estimating due to the load effect better performance could be achieved if the vehicle keeps attached to B1, the handover is delayed until the condition changes. For the sake of simplicity, we considered unidirectional video streaming traffic from the network to the vehicles and emulated the background traffic between A2 and the switch by defining the delay between them as 5 ms.

Figure 7b presents the obtained results in terms of latency for two cases. For Case 1 of no slice federation, latency significantly increases when the vehicle handoffs towards A2, at time instant 120 s. On the other hand, slice federation (Case 2) yields lower and stable latency and jitter values when the vehicle is kept attached to B1.

## 7. Conclusions

This paper presents an architectural framework specifically conceived to support V2X network slices, along with the description of the main mechanisms and enablers. The architecture relies on the closely-knit interactions of network softwarization technologies such as SDN, NFV and MEC.

The focus is on the design of a V2X slice for cooperative driving services and the behaviors entailed to enable them under intra- and inter-operator mobility. Although the benefits of network slicing for V2X can be sound in theory, practical validation is a necessary step that may include unanticipated critical implementation and deployment issues. Exploiting the fast prototyping and experimentation approach of Mininet-WiFi, in this study, we made some practical steps that hopefully can be useful to the wider research community. The conducted PoC experiments point to viable deployability options and potential benefits of the proposed framework and call for conducting more extensive evaluation campaigns.

We recognize that the complexity of the addressed topic entails further research efforts. Technical challenges upfront include more detailed implementation of the air interface protocols, the APIs definition between the conceived modules and strenuous inter-operator orchestration mechanisms, all of which are part of our research roadmap. In particular, future works will target the extension of the proposed framework for the design of near-seamless service migration strategies between MEC servers of different operators and their interplay with handover operations between edge-empowered RAN nodes.

## Figures and Tables

**Table 1:** Requirements and enablers for V2X network slicing.

Requirements/Features	Enablers
Multiple slices/sub-slices activation	NSSAI with SST and SD plus additional parameters (e.g., Device type)
Multi-tenancy management	Flexible slice template and description for different automotive verticals (e.g., road authorities, car manufacturers)
Massive communications	Multiple AMF instances to manage the signaling load due to mobility
Intra-operator mobility	Slice reconfiguration; MEC-assisted service migration; mobility prediction mechanisms
Inter-operator mobility	Basic set of functionalities for V2X slices agreed among all operators; unlicensed bandwidth for V2V communications in absence of infrastructure
